# Sepsis-Related Cardiomyopathy: Advances in Diagnosis and Treatment

**DOI:** 10.31083/RCM37044

**Published:** 2025-10-20

**Authors:** Bo Sun, Fenzan Wu, Tianqing Zhang

**Affiliations:** ^1^Department of Critical Care Medicine, Cixi People Hospital Medical Health Group (Cixi People Hospital), 315300 Cixi, Zhejiang, China; ^2^Department of Emergency Medicine, Second Affiliated Hospital of School of Medicine, Zhejiang University, 310052 Hangzhou, Zhejiang, China

**Keywords:** sepsis-induced cardiomyopathy, myocardial dysfunction, systemic inflammation, mitochondrial dysfunction, diagnostic biomarkers, treatment strategies

## Abstract

Sepsis-induced cardiomyopathy (SICM) constitutes a critical myocardial impairment that substantially elevates mortality risk in septic patients. The structural and functional changes of SICM are accompanied by metabolic disturbances, including mitochondrial dysfunction and altered myocardial metabolism. Diagnostic advancements feature biomarkers, circulating microRNAs, and imaging tools, such as speckle-tracking echocardiography. Therapies extend from vasopressors to immunomodulators, mitochondrial-targeted antioxidants, and mesenchymal stem cell-based approaches. Despite progress, challenges related to heterogeneity and the need for long-term data persist. Thus, integrated approaches leveraging machine learning and omics are essential for optimizing personalized care and reducing the global burden of SICM.

## 1. Introduction

Sepsis-induced cardiomyopathy (SICM) is a severe complication of sepsis characterized by transient cardiac dysfunction, including reduced ejection fraction and impaired ventricular function [1, 2]. It plays a significant role in the high mortality associated with sepsis. Globally, the prevalence of SICM among septic patients varies widely, with estimates ranging from 10% to 70% [3]. This variability is influenced by differences in diagnostic criteria, patient populations, and study methodologies.

In Europe and North America, multicenter studies highlight the clinical significance of SICM, associating it with prolonged intensive care unit (ICU) stays, higher healthcare costs, and elevated hospital mortality rates compared to septic patients without cardiac dysfunction [4, 5]. Similarly, studies from the Asia-Pacific region, including Australia and Japan, report comparable prevalence rates, further emphasizing the global clinical burden of SICM [6, 7]. Notably, disparities in clinical outcomes across different regions underline the necessity for standardized diagnostic criteria and universally applicable treatment protocols.

In low- and middle-income countries (LMICs), SICM poses distinct challenges due to resource limitations and disparities in healthcare infrastructure. Data from South Asia and Sub-Saharan Africa indicate a higher mortality burden associated with SICM, reflecting barriers to early diagnosis and limited access to advanced therapeutic interventions [8]. Addressing these global disparities requires an integrated approach involving improved diagnostic capabilities, cost-effective treatment strategies, and international cooperation.

At the same time, more and more studies have explored a broader range of SICM targeted therapies, but there has been no major clinical progress in the past decade. In this context, stem cells are increasingly becoming an attractive cell therapy in experimental and clinical models [9]. Preclinical data suggest that these cells and their secretion play a cornerstone role in controlling host immune responses. Host derived factors released from infected cells as well as pathogen associated molecular patterns can activate perivascular associated stem cells, thereby affecting the expression of cytokines/chemokines and growth factors for immune cell recruitment and stem cell mobilization [10].

This review aims to provide an updated, comprehensive overview of the mechanisms, diagnostic advances, and therapeutic innovations related to SICM, incorporating insights from international research and clinical experiences to address ongoing challenges and suggest future directions for global management improvements.

## 2. Theoretical Background

### 2.1 Definition and Classification of Sepsis-Induced Cardiomyopathy

Sepsis-induced cardiomyopathy is defined as reversible myocardial dysfunction occurring in the context of sepsis, characterized by reduced left ventricular ejection fraction (LVEF), impaired ventricular contractility, and/or diastolic dysfunction. Despite its transient nature, SICM significantly increases the risk of mortality, particularly in patients with pre-existing cardiovascular diseases [11]. Echocardiography remains the primary tool for SICM diagnosis, with advanced techniques such as strain imaging providing deeper insights into subclinical dysfunction [12, 13, 14, 15].

Although there is currently no universally accepted consensus definition for SICM, commonly proposed diagnostic criteria include: (1) confirmed presence of sepsis; (2) no prior history of primary structural heart disease; (3) new onset of acute cardiac dysfunction, such as reduced left ventricular ejection fraction (typically <50%) or abnormal myocardial strain patterns, or evidence of diastolic dysfunction (e.g., elevated E/e^′^ ratio, abnormal tissue Doppler imaging), acknowledging that ejection fraction alone may be misleading in the setting of reduced afterload and vasoplegia; and (4) exclusion of other identifiable causes of cardiac dysfunction, such as coronary artery disease, myocarditis, or severe valvular pathology [5, 14, 16].

Clinically, differential diagnosis should consider conditions such as acute coronary syndrome, viral myocarditis, and stress-induced cardiomyopathy (Takotsubo syndrome). These conditions typically require coronary angiography, virological testing, cardiac magnetic resonance imaging (CMR), and comprehensive patient history evaluation for accurate differentiation.

In Table 1 compares SICM with Takotsubo cardiomyopathy, viral myocarditis, and acute coronary syndrome (ACS) across key clinical dimensions, including onset pattern, LVEF, diastolic function (e.g., E/e^′^ ratio), cardiac biomarkers, and findings from coronary angiography. The purpose is to highlight distinguishing features that assist clinicians in accurate diagnosis and differential evaluation of acute cardiac dysfunction in the context of systemic illness.

**Table 1.** **Differential diagnosis of sepsis-induced cardiomyopathy (SICM) and other acute cardiac conditions**.

Feature	SICM	Takotsubo cardiomyopathy	Viral myocarditis	ACS
Onset pattern	Acute, infection-associated	Acute, often post-stress event	Acute or subacute	Acute chest pain onset
LVEF	Decreased, typically reversible	Decreased, apical dysfunction	Decreased, impaired	Significant elevation of troponin
Diastolic function	Often impaired, elevated E/e^′^	May show diastolic dysfunction	Commonly impaired	May be normal or impaired early
Coronary angiography	Normal	Normal	Normal or mild abnormalities	Presence of obstructive lesions

ACS, acute coronary syndrome; LVEF, left ventricular ejection fraction.

### 2.2 Pathophysiology of Sepsis-Induced Cardiomyopathy

The pathophysiology of SICM involves a multifactorial interplay of systemic inflammation, mitochondrial dysfunction, endothelial impairment, and metabolic derangements [17, 18, 19]. Emerging evidence continues to provide new insights into the mechanisms driving myocardial injury and dysfunction in SICM.

#### 2.2.1 Immune Dysregulation and Myocardial Inflammation

Sepsis-induced cardiomyopathy is fundamentally driven by an exaggerated immune response [20, 21, 22]. The release of pro-inflammatory cytokines such as tumor necrosis factor-alpha (TNF-α), interleukin (IL)-1β, and IL-6 disrupts myocardial contractility through alterations in calcium handling and beta-adrenergic receptor signaling [23, 24, 25]. Additionally, activation of Toll-like receptors (TLRs) and nuclear factor kappa-light-chain-enhancer of activated B cells (NF-κB) pathways further amplifies inflammation, leading to direct myocardial injury [26, 27, 28]. Persistent immune dysregulation often shifts into an immunosuppressive state, characterized by elevated levels of anti-inflammatory mediators such as IL-10, which exacerbate secondary infections and impair recovery [29, 30].

Recent studies have identified new molecular mediators of inflammation in SICM. For example, alarmins such as high mobility group box-1 (HMGB1) and S100 proteins have been implicated in promoting sustained inflammation and cardiac dysfunction [31, 32]. Targeting these mediators with specific inhibitors has shown promise in preclinical models, suggesting potential therapeutic avenues.

#### 2.2.2 Endothelial Dysfunction and Microvascular Impairment

Endothelial dysfunction plays a pivotal role in the pathogenesis of SICM by impairing oxygen delivery and promoting microvascular thrombosis [33, 34]. Loss of endothelial glycocalyx integrity, as evidenced by elevated syndecan-1 levels, correlates with increased vascular permeability, tissue edema, and myocardial ischemia [35]. Furthermore, endothelial activation triggers the coagulation cascade, leading to disseminated intravascular coagulation (DIC) and further compromising cardiac perfusion [36].

Nitric oxide (NO) dysregulation is another hallmark of endothelial dysfunction in SICM [37]. Excessive inducible NO synthase (iNOS) activity results in overproduction of NO, leading to vasodilation, hypotension, and decreased myocardial perfusion [38]. Conversely, impaired endothelial NO production contributes to reduced vascular reactivity and microvascular obstruction [39]. Therapies aimed at restoring glycocalyx integrity and modulating NO bioavailability are under investigation and have shown potential to improve cardiac outcomes in experimental models [40, 41].

#### 2.2.3 Mitochondrial Dysfunction and Oxidative Stress

Mitochondrial dysfunction is a hallmark of SICM, characterized by impaired oxidative phosphorylation and increased production of reactive oxygen species (ROS) [42, 43, 44]. This dysfunction plays a critical role in the energy crisis that underlies impaired myocardial contractility and cellular damage.

Recent studies highlight several key mechanisms:

Energy deficits: Reduced mitochondrial ATP production leads to insufficient energy for myocardial contraction and ion homeostasis [45]. Oxidative stress: Elevated ROS levels contribute to oxidative damage of lipids, proteins, and nucleic acids, exacerbating myocardial injury [46, 47]. Impaired quality control: Dysregulation of mitochondrial quality control processes, including mitophagy and biogenesis, results in the accumulation of damaged mitochondria, perpetuating dysfunction. Calcium handling abnormalities: ROS and dysfunctional mitochondria disrupt calcium homeostasis, further impairing contractility [48].

Emerging therapeutic strategies focus on preserving mitochondrial function and mitigating oxidative damage. For instance, mitochondrial-targeted antioxidants like MitoQ and SS-31 have demonstrated efficacy in reducing ROS production and improving myocardial performance in preclinical models [49]. Additionally, interventions aimed at enhancing mitophagy, such as pharmacological activators of PTEN-induced putative kinase 1 (PINK1)/Parkin pathways, show promise in restoring mitochondrial homeostasis [50].

Recent findings also emphasize the role of mitochondrial dynamics—fusion and fission processes—in SICM pathophysiology. Disrupted balance between these processes contributes to mitochondrial fragmentation and functional impairment. Therapies targeting proteins involved in mitochondrial dynamics, such as dynamin-related protein 1 (Drp1) inhibitors, are under investigation as potential treatments for SICM [51].

#### 2.2.4 Metabolic Reprogramming

Sepsis-induced metabolic reprogramming significantly impacts myocardial function [52]. Alterations in energy substrate utilization, including reduced fatty acid oxidation and increased glycolysis, contribute to metabolic inefficiency [53]. Elevated circulating lactate levels, a hallmark of sepsis, reflect impaired mitochondrial respiration and correlate with increased mortality risk [54, 55, 56]. Therapeutic strategies aimed at restoring metabolic balance, such as pyruvate dehydrogenase kinase inhibitors, are currently being explored [57, 58].

## 3. Advances in Diagnosis

Biomarkers play a critical role in diagnosing SICM and differentiating it from other cardiac conditions [59]. Elevated levels of high-sensitivity troponins and brain natriuretic peptide (BNP) are commonly observed in SICM [60]. Recent advancements have identified novel biomarkers, such as soluble suppression of tumorigenesis-2 (sST2) and growth differentiation factor-15 (GDF-15), which have shown promise in distinguishing SICM from other causes of myocardial injury [61]. Additionally, combining traditional markers with emerging biomarkers enhances diagnostic accuracy and prognostic assessment [62].

## 4. Therapeutic Innovations

In Table 2 summarizes current therapeutic approaches for sepsis-induced cardiomyopathy according to their stage of clinical implementation. Routine clinical use includes well-established treatments widely utilized in standard practice. Clinical trial indicates therapies under active evaluation, with ongoing studies assessing their efficacy and safety in SICM patients.

**Table 2.** **Clinical application status of therapies for sepsis-induced cardiomyopathy (SICM)**.

Therapeutic category	Specific methods	Stage of clinical application
Hemodynamic support	Norepinephrine	Routine clinical use
Hemodynamic support	Vasopressin	Routine clinical use
Hemodynamic support	Dobutamine	Routine clinical use
Hemodynamic support	Angiotensin II (NCT02338843)	Routine clinical use/Clinical trial
Hemodynamic support	Esmolol (NCT01231698)	Routine clinical use
Hemodynamic support	Landiolol (NCT04931225)	Clinical trial
Blood purification therapies	Polymyxin B Hemoperfusion	Routine clinical use
Blood purification therapies	CytoSorb Cytokine Adsorption (NCT04391920)	Clinical trial
Stem cell therapies	Mesenchymal stem cells (MSCs) (NCT02883803)	Clinical trial
Exosome therapies	MSC or M2 macrophage-derived exosomes	Clinical trial
Immunomodulatory therapies	Interleukin (IL)-1 receptor antagonist (Anakinra)	Clinical trial
Immunomodulatory therapies	IL-6 receptor antagonist (Tocilizumab) (NCT04320615)	Clinical trial
Immunomodulatory therapies	TNF-α inhibitors (Etanercept, Infliximab)	Clinical trial
Mitochondrial-targeted therapies	MitoQ, SS-31, Coenzyme Q10	Preclinical/Early-phase clinical research
Adjunctive therapies	Vitamin C	Clinical trial
Adjunctive therapies	Thiamine (NCT03540628)	Clinical trial
Adjunctive therapies	Selenium	Clinical trial
Mechanical circulatory support	Extracorporeal membrane oxygenation (ECMO)	Routine clinical use

Clinical trial registration available at https://clinicaltrials.gov.

### 4.1 Hemodynamic Support

Hemodynamic stabilization is fundamental in the management of SICM, aiming to optimize cardiac output, ensure adequate organ perfusion, and counteract the adverse effects of systemic inflammation on cardiovascular function. Vasopressors and inotropes remain the cornerstone of hemodynamic support, while advancements in therapeutic strategies and monitoring techniques have enhanced precision and effectiveness in this critical area.

Norepinephrine is the first-line vasopressor for restoring vascular tone and maintaining mean arterial pressure (MAP), typically targeting a threshold of 65 mmHg or higher [63]. In cases of refractory shock, vasopressin is frequently employed as a second-line agent to achieve hemodynamic goals while minimizing catecholamine exposure. The VASST trial highlighted improved outcomes in specific septic shock subgroups treated with vasopressin [64]. Dobutamine, an inotropic agent, is commonly used to enhance myocardial contractility, particularly in patients with reduced ejection fraction or persistent hypoperfusion despite preload optimization. Advanced monitoring techniques, such as pulmonary artery catheterization and echocardiography, guide the appropriate use of inotropes, minimizing complications like tachyarrhythmias [65].

In addition to conventional agents, alternative pharmacologic therapies have shown promise in improving cardiac performance and hemodynamic stability in SICM.

Levosimendan, a calcium sensitizer and potassium ATP channel opener, enhances myocardial contractility without increasing intracellular calcium levels, thereby minimizing arrhythmogenic risk. It also improves peripheral perfusion via vasodilatory effects. Clinical trials and meta-analyses suggest levosimendan may augment cardiac output and reduce vasopressor dependence in septic shock patients, though mortality benefits remain inconclusive [66, 67, 68]. Off-label use in SICM is increasingly explored, but patient selection and optimal dosing strategies require further validation.

Dexmedetomidine, a selective α2-adrenergic agonist used for ICU sedation, exhibits cardioprotective and sympatholytic effects. By attenuating sympathetic tone and reducing catecholamine surge, it improves myocardial oxygen balance and may mitigate SICM-related tachycardia and inflammation. Retrospective and prospective studies have demonstrated reduced mortality and improved myocardial function in sepsis patients receiving dexmedetomidine [69, 70]. Nevertheless, cautious titration is essential to avoid bradycardia or hypotension in unstable patients.

Emerging hemodynamic strategies offer additional avenues for optimization. Dynamic fluid responsiveness monitoring, employing technologies like pulse pressure variation (PPV) and stroke volume variation (SVV), allows personalized fluid resuscitation, reducing the risk of fluid overload and secondary complications such as pulmonary edema. Adjunctive therapies, including angiotensin II and terlipressin, have shown promise in managing refractory hypotension. Angiotensin II, as demonstrated in the ATHOS-3 trial, rapidly stabilizes blood pressure in patients resistant to conventional vasopressors [71]. Terlipressin, with its vasoconstrictive properties, has demonstrated potential in reducing mortality in selected septic shock patients [72].

β-blockade has been proposed as a novel strategy for managing septic tachycardia. Esmolol, a short-acting beta-blocker, has been studied for its potential benefit in controlling heart rate and improving left ventricular filling time. However, the ESMO SEPSIS trial demonstrated that in the very early phase of septic shock, rapid heart rate reduction by fast esmolol titration may actually increase the risk of hypotension and reduce cardiac index, despite maintaining adequate tissue perfusion [68]. Moreover, a recent systematic review and meta-analysis evaluating the effects of ultra-short-acting beta-blockers (esmolol or landiolol) reported no clear mortality benefit among septic patients with persistent tachycardia. Importantly, the study highlighted significant heterogeneity, as outcomes differed notably between single-center and multicenter randomized controlled trials [69].

To refine patient selection for β-blockade therapy, emerging evidence suggests that specific hemodynamic parameters may help distinguish patients likely to benefit from those at risk of harm. In particular, a study by Morelli *et al*. [73] demonstrated that baseline hemodynamic profiles, including cardiac index, systemic vascular resistance, and lactate levels, could predict responsiveness to esmolol therapy. Patients with preserved preload reserve and adequate cardiac performance were more likely to tolerate and benefit from β-blockade without compromising tissue perfusion.

These findings emphasize caution in the routine clinical use of β-blockade therapy in septic shock, suggesting that patient selection, precise timing, careful hemodynamic monitoring, and careful titration remain critical to optimizing potential benefits and minimizing risks.

Integrating artificial intelligence (AI) with hemodynamic monitoring systems presents a promising avenue for precision management. Algorithms leveraging real-time data from PPV, SVV, and other dynamic variables enable the prediction of fluid responsiveness and the tailoring of therapeutic interventions [74]. Future directions include the development of personalized hemodynamic targets based on genetic predispositions, biomarker profiles, and advanced imaging techniques. Innovations in non-invasive monitoring, such as wearable biosensors, offer significant potential for improving patient outcomes, particularly in resource-limited settings.

### 4.2 Extracorporeal Blood Purification Therapies

Extracorporeal blood purification therapies have increasingly garnered attention in the management of SICM. Among these, polymyxin B hemoperfusion (PMX-HP) stands out as a specialized therapy, selectively adsorbing endotoxins (lipopolysaccharides, LPS) from circulating blood, thus attenuating the systemic inflammatory cascade triggered by endotoxemia. The primary mechanism by which PMX-HP exerts its cardioprotective effect involves the reduction of pro-inflammatory cytokines such as TNF-α, IL-1β, and IL-6, stabilization of hemodynamic parameters, and potential mitigation of myocardial injury.

Several clinical studies have demonstrated that PMX-HP significantly improves hemodynamic stability, reflected by increases in MAP, reduced vasopressor requirements, and decreased levels of circulating inflammatory biomarkers [75]. For example, Vincent *et al*. [76] reported improved MAP and vasopressor response in septic shock patients undergoing PMX-HP. More recently, Cutuli *et al*. [77] observed reductions in cardiac biomarkers such as troponin and N-terminal pro B-type natriuretic peptide (NT-proBNP), suggesting a potential role in limiting myocardial injury.

However, it is crucial to acknowledge existing controversies concerning the clinical efficacy of PMX-HP therapy, as some randomized controlled trials have yielded mixed results, indicating variability related to patient selection, timing of intervention, and specific clinical settings. Further large-scale, randomized clinical studies are warranted to clearly define the patient populations that may benefit most from this therapy and to elucidate its definitive role in clinical practice.

### 4.3 Immunomodulatory Therapies

Immunomodulatory therapies targeting the dysregulated immune response in SICM offer a promising avenue for treatment. These therapies aim to restore the balance between the hyperinflammatory and immunosuppressive phases of SICM, mitigating cytokine storms while preventing immune exhaustion. By addressing key inflammatory pathways, these approaches provide a foundation for advancing SICM management.

Cytokine inhibition remains a cornerstone of these strategies, with agents such as anakinra, an IL-1 receptor antagonist, demonstrating the ability to reduce myocardial inflammation and improve cardiac function. Clinical trials have shown that anakinra lowers mortality rates and enhances organ function in sepsis patients with elevated IL-1β levels, though its specific efficacy in SICM requires further exploration [75]. Similarly, tocilizumab, an IL-6 receptor antagonist, has shown potential in mitigating the inflammatory cascade, improving myocardial recovery in preclinical models of septic shock [76]. In contrast, TNF-α inhibitors, such as etanercept and infliximab, have delivered mixed results, reflecting the complexity of modulating this pivotal cytokine in the inflammatory response [78].

Immune checkpoint inhibitors have emerged as a novel approach, targeting pathways such as programmed death-1 (PD-1) and cytotoxic T-lymphocyte-associated protein 4 (CTLA-4). These therapies restore immune cell function and reduce secondary infections, although their direct effects on myocardial recovery remain under investigation [79].

Cytokine adsorption and hemoadsorption therapies represent a complementary approach, aiming to remove excess inflammatory mediators from circulation. Devices such as CytoSorb have shown promise in stabilizing hemodynamics and reducing cytokine levels, leading to improved cardiac function. Additionally, polymyxin B hemoperfusion, targeting endotoxin removal, may indirectly reduce myocardial inflammation by limiting the release of inflammatory mediators. However, mixed results from trials such as EUPHRATES underscore the importance of patient selection for these interventions [80].

Adaptive immunomodulation strategies offer further potential by selectively enhancing protective immune responses. Granulocyte-macrophage colony-stimulating factor (GM-CSF) has been shown to restore monocyte function, improving immune resilience and reducing mortality in SICM models [81]. IL-7, a T-cell growth factor, holds promise in reversing lymphopenia and enhancing immune surveillance, with early studies reporting improvements in lymphocyte counts and reductions in secondary infections [82].

Emerging technologies, including single-cell RNA sequencing and transcriptomics, enable precision immunotherapy by identifying patient-specific immune profiles. These insights facilitate the development of targeted interventions, allowing for more personalized and effective treatment strategies. For example, transcriptomic analyses have revealed the upregulation of specific immune checkpoint genes in SICM, providing novel targets for therapeutic intervention [83].

Incorporating these immunomodulatory therapies into SICM treatment paradigms requires further research to refine patient selection, optimize timing, and explore combination strategies. Such efforts will pave the way for more tailored and effective interventions, ultimately improving outcomes in this challenging condition.

### 4.4 Mitochondrial-Targeted Therapies

Mitochondria play a central role in the pathophysiology of SICM, making them a critical target for therapeutic interventions. Mitochondrial dysfunction in SICM is characterized by impaired oxidative phosphorylation, excessive production of ROS, and disrupted mitophagy, all of which contribute to myocardial energy deficits and cellular injury. Addressing these dysfunctions has become a cornerstone in efforts to mitigate myocardial damage and improve patient outcomes.

Inflammatory cytokines often inhibit mitochondrial complexes I and III of the electron transport chain, leading to reduced ATP production and exacerbating myocardial energy deficits [84]. Concurrently, excessive ROS production damages mitochondrial DNA, proteins, and lipids, creating a vicious cycle of oxidative stress and mitochondrial dysfunction. This oxidative damage activates pro-apoptotic pathways, such as cytochrome c release, further contributing to myocardial cell death. Impaired mitophagy, due to disruptions in the PINK1/Parkin pathway, results in the accumulation of dysfunctional mitochondria, amplifying energy deficits and oxidative damage [85]. Compounding these challenges, mitochondrial biogenesis is often suppressed in SICM, with downregulation of peroxisome proliferator-activated receptor gamma coactivator (PGC)-1α hindering the regeneration of functional mitochondria and delaying myocardial recovery [86]. Mitochondrial-targeted therapies aim to address these pathological processes through innovative approaches. Mitochondrial antioxidants, such as MitoQ and SS-31, neutralize ROS and stabilize mitochondrial membranes. Stimulating mitochondrial biogenesis using drugs such as bezafibrate promotes the regeneration of functional mitochondria, improving left ventricular function and overall energy homeostasis in SICM models [87].

Other strategies focus on stabilizing the electron transport chain to preserve ATP synthesis and minimize ROS production. For example, agents like EPI-743 and idebenone have shown potential in stabilizing mitochondrial activity under septic conditions, thereby reducing oxidative damage [88]. Additionally, coenzyme Q10 supplementation has emerged as a viable therapeutic option, serving as a vital component of the electron transport chain and an endogenous antioxidant. Clinical trials have reported reductions in myocardial injury markers and improvements in hemodynamic parameters with Coenzyme Q10 therapy [89]. Targeting the mitochondrial permeability transition pore (mPTP) is another promising avenue. mPTP inhibitors, such as cyclosporine A, prevent mitochondrial depolarization and calcium overload, protecting against mitochondrial dysfunction and cardiomyocyte death in SICM [90]. These approaches not only improve mitochondrial stability but also enhance overall cardiac function.

Despite these advances, challenges remain in translating mitochondrial-targeted therapies into clinical practice. The heterogeneity in mitochondrial dysfunction across SICM patients necessitates personalized therapeutic approaches based on reliable biomarkers of mitochondrial health. Drug delivery is another obstacle, as ensuring targeted delivery to cardiac tissues remains difficult. Advances in nanotechnology-based delivery systems, such as lipid nanoparticles, hold promise in enhancing drug bioavailability and tissue specificity [91].

Future research should focus on identifying biomarkers to assess mitochondrial function in real time, facilitating early diagnosis and therapeutic monitoring. Combining mitochondrial-targeted therapies with other interventions, such as anti-inflammatory agents or vasopressors, may yield synergistic benefits. Additionally, the integration of omics technologies, including proteomics and transcriptomics, can further refine patient stratification and therapeutic targeting in SICM.

### 4.5 Stem Cell-Based Therapies

Stem cell-based therapies have emerged as a promising avenue for addressing SICM due to their regenerative, anti-inflammatory, and immunomodulatory properties. These therapies aim to restore myocardial function, reduce systemic inflammation, and promote tissue repair, addressing the multifactorial pathophysiology of SICM.

Mesenchymal stem cells (MSCs) are among the most extensively studied cell types for SICM treatment. MSCs exert potent immunomodulatory effects by releasing anti-inflammatory cytokines, such as IL-10 and transforming growth factor-beta (TGF-β), while suppressing pro-inflammatory cytokines like TNF-α and IL-6. Additionally, MSCs influence macrophage polarization, shifting them from the pro-inflammatory M1 phenotype to the reparative M2 phenotype, which is critical for tissue healing [92]. Beyond their immunomodulatory roles, MSCs release extracellular vesicles and exosomes containing bioactive molecules, including microRNAs, proteins, and growth factors. These vesicles contribute to mitochondrial function restoration, oxidative stress reduction, and enhanced angiogenesis, thereby promoting myocardial repair [93].

Angiogenesis is another vital aspect of stem cell therapy in SICM. Growth factors such as vascular endothelial growth factor (VEGF) and fibroblast growth factor (FGF), secreted by MSCs, stimulate endothelial cell proliferation and repair, improving microvascular perfusion and myocardial oxygenation microvascular perfusion and myocardial oxygenation [94]. Although the direct differentiation of stem cells into cardiomyocytes is limited, induced pluripotent stem cells (iPSCs) offer a unique advantage. iPSCs can generate patient-specific functional cardiomyocytes, showing potential in reversing myocardial damage in preclinical study [95].

Delivery methods for stem cell therapies have been tailored to enhance therapeutic efficacy. Intravenous infusion is the most commonly used approach due to its simplicity, but it is often limited by cell entrapment in the lungs. Intramyocardial injection, delivering stem cells directly into myocardial tissue, improves localization and therapeutic impact, especially in preclinical studies. Alternatively, intracoronary infusion allows targeted delivery to the myocardium via the coronary arteries, ensuring higher retention rates and better myocardial perfusion. Exosome-based delivery systems have gained attention as a safer, cell-free alternative, leveraging the regenerative potential of stem cell-derived exosomes while mitigating risks like immune rejection and tumorigenesis [96].

Preclinical and clinical studies provide robust evidence for the safety and efficacy of stem cell-based therapies in SICM. Animal models treated with MSCs demonstrated reduced myocardial apoptosis, decreased inflammatory cytokines, and improved cardiac contractility. Similarly, early-phase clinical trials reported enhanced hemodynamic parameters, reduced inflammation, and shorter ICU stays in septic patients receiving MSC therapies [97].

### 4.6 Adjunctive Therapies

Adjunctive therapies play a vital role in managing SICM by targeting specific pathophysiological mechanisms, including oxidative stress, immune dysregulation, and mitochondrial dysfunction. These therapies complement standard care strategies, aiming to reduce inflammation, enhance myocardial function, and mitigate systemic complications.

Vitamin C, a potent antioxidant, has gained considerable attention for its ability to mitigate oxidative stress and stabilize endothelial function. By scavenging ROS and regenerating endogenous antioxidants like glutathione, vitamin C reduces cellular damage in myocardial tissues. It also decreases endothelial permeability by stabilizing the glycocalyx and enhances nitric oxide production, improving vascular tone and myocardial oxygen delivery. Clinical trials, such as the CITRIS-ALI study, have demonstrated that high-dose intravenous vitamin C reduces organ failure scores in septic patients, although its effects on mortality remain inconclusive [98]. One published research, including the LOVIT trial, seeks to refine dosing protocols and identify patient subgroups that may derive the greatest benefit [99, 100]

Thiamine, an essential coenzyme in cellular metabolism, addresses mitochondrial dysfunction by facilitating the conversion of pyruvate to acetyl-CoA in the Krebs cycle. This restoration of oxidative metabolism reduces lactate accumulation and alleviates metabolic acidosis, common features in SICM. A study has shown that thiamine supplementation decreases lactate levels and improves survival in septic patients with lactic acidosis [101]. When combined with vitamin C and hydrocortisone, thiamine has demonstrated synergistic effects, improving hemodynamic parameters and reducing mortality rates [101].

Selenium, a critical micronutrient and cofactor for glutathione peroxidase, plays a significant role in antioxidant defense. By neutralizing ROS and modulating immune responses, selenium reduces myocardial oxidative damage and supports cellular repair. While randomized trials have shown that selenium supplementation reduces oxidative stress markers and improves survival rates in septic patients, conflicting results in subsequent studies highlight the need for further investigation to establish its definitive role in SICM [102].

Omega-3 fatty acids, such as eicosapentaenoic acid (EPA) and docosahexaenoic acid (DHA), offer cardioprotective benefits through their anti-inflammatory and membrane-stabilizing properties. These fatty acids modulate inflammatory pathways by inhibiting the production of pro-inflammatory eicosanoids and promoting the synthesis of specialized pro-resolving mediators (SPMs). They also enhance membrane fluidity in cardiomyocytes, improving myocardial contractility and reducing arrhythmias. Clinical studies have reported improved cardiac function and reduced inflammatory markers in septic patients receiving omega-3 fatty acid supplementation [103].

Despite their potential benefits, the efficacy of adjunctive therapies is influenced by patient-specific factors, including baseline oxidative stress levels, immune status, and metabolic reserves. Identifying reliable biomarkers, such as lactate levels and oxidative stress markers, is essential for guiding therapy selection and optimizing outcomes. Additionally, variability in dosing regimens and treatment protocols remains a challenge, underscoring the need for standardized guidelines informed by robust clinical evidence.

Looking forward, combining adjunctive therapies with standard treatments, such as vasopressors and inotropes, may yield synergistic benefits by addressing multiple aspects of SICM pathophysiology. Future research should focus on refining these combinations and exploring novel adjunctive agents to further improve patient outcomes.

### 4.7 Exosome-Based Therapies

Exosomes, nano-sized extracellular vesicles derived from various cell types, have emerged as a promising therapeutic tool for SICM. By facilitating intercellular communication, exosomes transfer bioactive molecules, including RNAs, proteins, and lipids, to recipient cells, thereby modulating inflammatory responses, promoting angiogenesis, and enhancing mitochondrial repair. Their natural stability, biocompatibility, and specificity position exosomes as an innovative approach in both diagnostics and therapeutics for SICM.

Exosomes derived from MSCs and M2 macrophages are particularly promising due to their anti-inflammatory and reparative properties. MSC-derived exosomes carry microRNAs, such as miR-21 and miR-146a, which suppress pro-inflammatory mediators like TNF-α and IL-6, reducing systemic inflammation. Additionally, these exosomes enhance mitochondrial function by delivering components that mitigate oxidative stress and restore mitochondrial dynamics. A preclinical study has demonstrated that MSC-derived exosomes improve cardiac function, reduce myocardial fibrosis, and enhance left ventricular ejection fraction in SICM models [104].

M2 macrophage-derived exosomes have shown significant potential in promoting cardiac repair through their ability to modulate immune responses. These exosomes facilitate the transition of macrophages from the pro-inflammatory M1 phenotype to the reparative M2 phenotype, thereby reducing systemic inflammation. They also promote angiogenesis by delivering pro-angiogenic factors, such as VEGF and angiopoietin-1, which improve microvascular perfusion and support myocardial recovery [105].

Cardiac-specific exosomes, derived from healthy cardiomyocytes or engineered cardiac progenitor cells, offer a targeted therapeutic approach. These exosomes deliver mitochondrial repair components, including mitochondrial RNAs and proteins, directly to damaged cardiomyocytes, restoring mitochondrial respiration and reducing oxidative damage. Moreover, they enhance calcium handling by transferring proteins such as Sarcoplasmic/Endoplasmic Reticulum Ca^2+^-ATPase 2a (SERCA2a), which improves myocardial onlzand overall cardiac function [106].

Efforts to optimize exosome-based therapies have focused on engineering and delivery methods. Genetic modification of exosome-producing cells enables the enrichment of therapeutic cargo, such as overexpressed VEGF or specific microRNAs. Surface functionalization, including the addition of targeting ligands, enhances the specificity of exosome delivery to injured myocardial tissues. Additionally, innovative loading techniques, such as electroporation and sonication, allow for the incorporation of therapeutic agents like small interfering RNAs (siRNAs) or drugs into exosomes, further enhancing their therapeutic potential [107].

Despite their promise, challenges remain in scaling up the production of high-purity exosomes while maintaining consistency and quality. Effective delivery to the myocardium without off-target effects is another critical barrier, necessitating advancements in nanotechnology-based carriers, such as lipid nanoparticles. Furthermore, the regulatory and safety considerations surrounding exosome therapies, including potential immune reactions and long-term effects, require thorough evaluation before widespread clinical application.

Future directions for exosome-based therapies include the development of standardized isolation and characterization techniques to ensure reproducibility. Large-scale randomized controlled trials are essential to validate preclinical findings and optimize therapeutic protocols. Exploring the combination of exosomes with other interventions, such as immunomodulators or mitochondrial-targeted therapies, may provide synergistic benefits. Advances in omics technologies and machine learning can further refine the design of personalized exosome therapies, maximizing their potential to improve outcomes in SICM.

## 5. Research Challenges and Future Directions

### 5.1 Translational Gaps

Bridging the gap between preclinical findings and clinical application remains a significant challenge in SICM research. The variability in animal models and the heterogeneity of septic patients complicate the translation of therapeutic approaches.

### 5.2 Economic and Ethical Considerations

The high cost of advanced diagnostics and therapies limits their accessibility, particularly in resource-limited settings. Ethical concerns regarding equitable distribution and the use of experimental treatments must be addressed through robust regulatory frameworks.

### 5.3 Global Collaboration

International partnerships and the establishment of SICM registries can facilitate data sharing, standardization of diagnostic criteria, and the development of globally applicable treatment protocols.

The evolution of SICM diagnosis and management reflects a remarkable journey from initial uncertainty to burgeoning precision. Over the past decade, advancements in our understanding of SICM’s pathophysiology—from immune dysregulation and endothelial dysfunction to mitochondrial damage—have transformed it from a poorly understood complication of sepsis to a targeted focus of critical care medicine.

Historically, SICM diagnosis relied on nonspecific markers and clinical observation. However, breakthroughs in biomarkers, such as sST2 and GDF-15, have revolutionized early detection, offering predictive value and specificity. Imaging advancements, including three-dimensional (3D) echocardiography and strain imaging, have further refined our ability to evaluate cardiac dysfunction, ensuring that subclinical abnormalities are not overlooked.

Therapeutic progress has been equally profound. From conventional inotropes like dobutamine to cutting-edge mitochondrial-targeted antioxidants such as SS-31, treatment options have diversified significantly. Emerging modalities like stem cell-based therapies and exosome-derived interventions promise not only to mitigate myocardial damage but also to facilitate long-term recovery. These innovations underscore a shift toward personalized medicine, leveraging patient-specific data from omics technologies and artificial intelligence to tailor interventions.

Despite these strides, critical challenges remain. Translational gaps persist, particularly in adapting preclinical findings for heterogeneous patient populations. Economic and logistical barriers continue to hinder widespread adoption of advanced diagnostics and treatments, especially in low-resource settings. Furthermore, ethical considerations surrounding the use of AI-driven tools and experimental therapies demand attention.

The trajectory of SICM management is promising. Unified international registries, cross-disciplinary collaborations, and equitable resource allocation will be pivotal in addressing existing gaps. As research accelerates and global cooperation strengthens, the future of SICM care appears increasingly bright.

The progress achieved thus far serves as a testament to the resilience and innovation of the medical community. From early detection to transformative therapies, SICM is evolving from a daunting clinical challenge into a manageable condition. This journey reminds us that with each diagnostic tool refined and therapeutic strategy optimized, we come closer to rewriting the narrative of SICM—one of hope, healing, and improved patient outcomes.

## 6. Conclusion

This review focuses on sepsis induced cardiovascular disease. Its pathological mechanisms include immune dysfunction and myocarditis, endothelial dysfunction and microvascular injury, mitochondrial dysfunction and oxidative stress, and metabolic reprogramming. At present, there are various treatment methods for SICM, including hemodynamic support, immune regulation therapy, mitochondrial targeted therapy, stem cell assisted therapy, and exosome therapy. However, the research on SICM faces the challenge of transitioning from preclinical to clinical application. In the future, it is necessary to improve the prognosis of patients through global cooperation, such as establishing registration centers. This review provides a brief overview of recent research insights on the adverse effects of sepsis on cardiovascular health. In the field of cardiology prevention and public health strategies, the prevention and treatment of sepsis induced cardiomyopathy should become an indispensable and important component.
